# Perceived usefulness of, engagement with, and effectiveness of virtual reality environments in learning industrial operations: the moderating role of openness to experience

**DOI:** 10.1007/s10055-023-00793-0

**Published:** 2023-04-24

**Authors:** Eugene Yin-cheung Wong, Ray Tak-yin Hui, Hao Kong

**Affiliations:** 1 Department of Supply Chain and Information Management, School of Decision Sciences, The Hang Seng University of Hong Kong, Hong Kong, China; 2 NUCB Business School, Nagaya University of Commerce and Business, Nagoya, Japan; 3 Department of Management, School of Business, The Hang Seng University of Hong Kong, Hong Kong, China

**Keywords:** Virtual reality, Pedagogical development, Openness to experience, Perceived usefulness of VR, Attitude towards learning, Technology acceptance model

## Abstract

The development of virtual reality (VR) in enhancing the effectiveness of the learning process, with its interactive, immersive, and intuitive pedagogical environment, has become a necessity for corporations with increasingly complex operations. However, VR users’ perceptions, openness and learning effectiveness are seldom comprehensively evaluated, particularly in learning complex industrial operations. In this study, grounded in the technology acceptance model, a moderated mediation model of perceived usefulness, ease of use, openness to experience, and engagement in VR-based learning was developed. The model was empirically validated using responses collected from 321 users who were trained on aircraft and cargo terminal operations powered by a novel VR-based learning platform. A survey to measure openness to experience and a pre-training performance test were carried out, followed by a post-training survey of learners’ intrinsic factors, including the influence of perceived usefulness, openness to experience, and attitude towards learning. The study revealed that learners with an open attitude towards experiencing new technology tend to perceive VR technology as a useful platform for training. In addition, the learners with more positive views of VR technology-supported training were more engaged in learning.

## Introduction

The importance of virtual reality (VR) in education is growing as immersive technology increases in popularity and availability, more learning content requiring VR support is identified (Hamilton, McKechnie, Luo et al. 2021), at the same time as the cost of equipment declines. VR products were selected as among the top inventions of 2016 (Time 2016), with technology leaders actively inventing and trademarking VR technologies. The number of studies on the use of VR and meta technologies in teaching and learning is increasing, boosting demand for development of hardware and software for visualisation and cooperation, as well as expectations (Kalantari and Rauschnabel 2018; Won et al. 2021; Xu et al. 2021; Xi et al. 2022). Rising demand for VR headsets meant over 14 million VR and augmented reality (AR) devices were sold in 2019, while the number of VR users has already surpassed 171 million worldwide (Petrov 2019). The price of VR devices available to the consumer has gradually decreased, and they are more widely available in retail stores. The global VR, AR, and mixed reality (MR) market reached US$28 billion in 2021, rising to over US$250 billion by 2028 (Alsop 2022a). The commercial use cases for VR and AR that are expected to receive the largest investment in 2024 are training with US$4.1 billion forecast to be invested in this field (Alsop 2022b).

The dramatic surge in the use of VR has extended to professional education and training. This is the result of the greater complexity of data and operation processes in organisations, tighter operational security and safety standards, and an increasing awareness that VR can be used for cultural preservation (Fussell and Truong 2021; Zhang et al. 2022). Traditional modes of teaching and learning, such as classroom lecturing, textbook reading, video learning, and case-study discussions, do not capture the complexity of certain operations. They are also often unable to demonstrate multi-dimensional operations and statistics (Ding et al. 2020). Large datasets with multiple dimensions require a sophisticated virtual environment for visualisation (Wong et al. 2020). Tightened security and safety guidelines often limit the accessibility of restricted areas for field trips and onsite visits to areas such as cargo terminals in the logistics and transport sector, clinical and surgical suites in hospitals, and trading platforms in finance. Research into the use of VR in education is therefore increasing, and disciplines including engineering (Abulrub et al. 2011; Rovira and Slater 2017; Soliman et al. 2021), medicine (Lohre et al. 2020; Izard et al. 2018), industrial operations (Bourhim and Cherkaoui 2020; Chen et al. 2019), arts (Parker and Saker 2020; Han 2015), and geometry (Kaufmann and Schmalstieg 2006) are being examined.

The successful adoption and implementation of VR in teaching rely on the audience’s acceptance and attitudes, as well as the perceived usefulness of the new technology. The technology acceptance model (TAM) has been used to study learners’ acceptance of VR (e.g. Chang et al. 2018a, b; Durodolu 2016; Fussell and Truong 2021; Zhang et al. 2022). Durodolu (2016) reviewed learners’ acceptance and use of new technology in instilling the skills needed for information literacy. Chang et al. (2018a, b) carried out a similar experiment to analyse users’ perceptions of VR and whether they intended to adopt a VR-based mental rotation-training system. The study found that users’ positive perceptions and intentions of using the system were amplified when better immersive and interactive experiences were provided. Fussell and Truong (2021) also adopted the TAM to explain and predict relationships between ease of use, usefulness, attitudes towards, and intentions regarding the use of AR for in-flight training, while Zhang et al. (2022) found that perceived usefulness and perceived ease of use directly predicted the attitudes of 1158 workers and students in the construction industry towards using VR technology. However, the majority of studies in the VR literature have focused mainly on the effect of the TAM on users’ intention to use VR, whereas very few have studied users’ actual learning (e.g. Zhang et al. 2017). To address this gap, this study aims to examine how the TAM influences the effectiveness of VR in learning.

To evaluate the acceptance and perceived usefulness of VR in teaching and learning, it is important to assess and analyse the effectiveness of VR in learning, especially where complex industrial procedures are concerned. Reznek et al. (2002) evaluated construct and content validity as well as learners’ perceptions of a VR intravenous insertion simulator in a training session with 41 users. The usefulness, ease of use and overall appeal of the simulator were addressed. Huang et al. (2010) attempted to investigate the effectiveness of learning using Web-based three-dimensional (3D) technologies with VR features and proposed guidelines for the effective use of VR in learning environments. Zhang et al. (2017) reviewed the distinct features of VR regarding visualisation, interaction, representational fidelity, and immediacy of control to improve users’ learning outcomes. A survey of 180 users was therefore conducted to evaluate the learning effectiveness of VR, which concluded that VR could influence reflective thinking and lead to further indirect improvement in perceived learning effectiveness. Similar studies were performed by Lee et al. (2009, 2010) and Jou and Wang (2013).

Based on the theory of reasoned action (TRA) (Ajzen and Fishbein 1980; Fishbein, and Ajzen. 1975), from which the TAM was adapted to apply to information systems, Devaraj and his colleagues (2008) argued that personality might be an external variable that interacts with TAM factors, such as perceived technology acceptance and ease of use, and thus affect users’ performance and behaviour. For example, Kober and Neuper (2013) found that users’ personalities, including aspects such as their imagination, perspectives, and immersive tendencies, were significantly correlated with their perceived presence in VR. Widyanti and Hafizhah (2021) showed that the Big Five personality traits, such as emotional stability and openness to experience, were significant for susceptibility to VR sickness but in different ways. However, research on the role of personality in VR-based learning remains scarce in the existing literature. To fill this gap, this study will examine the effect of users’ openness to experience—one of the most widely studied, learning-related personality attributes in the Big Five Inventory (Chow 2018; Costa and McCrae 1992)—on the TAM of VR technology, and users’ learning effectiveness.

Given the increasing complexity of operating aircraft and cargo terminals, learners are not able to fully understand the procedures with the use of traditional learning modes. Tighter security in these sites makes it more difficult for students, and even practitioners, to gain advanced technical knowledge or practise their skills. Therefore, a novel interactive, immersive, and intuitive VR learning environment that simulated an aircraft and cargo terminal is need is needed. The scene was developed to facilitate teaching and learning in the supply chain and logistics programmes. A pilot study was conducted to examine the interactive effects of openness to information technology (IT) and the perceived usefulness of VR training and attitudes towards it. After the pilot study, a further extensive and detailed study was carried out to gauge various factors, including openness to experience, perceived usefulness, perceived ease of use, engagement in VR-based learning and learning effectiveness.

## Virtual reality—from technology development to learning effectiveness

Inventions and experiments related to VR began in the 1950s. Heilig (1962) developed a mechanical device called a ‘Sensorama’, with 3D images, peripheral vision, and multiple senses, including sight, sound, smell, and touch. The computer scientist Ivan Sutherland developed a VR and AR head-mounted display in 1968, which was followed in 1978 by the ‘Aspen Movie Map’, a hypermedia and VR system from the Massachusetts Institute of Technology that showed a virtual simulation of Aspen, Colorado (Krueger et al. 1985). Lanier (1989) later developed various VR devices, such as the ‘Data Glove’, the ‘Eye Phone’, and the ‘Audio Sphere’. Cruz-Neira et al. (1992) developed the first cubic immersive room, the Cave Automatic Virtual Environment (CAVE). This is a hollow cube with display-screens surrounding the viewer, who moved within the CAVE. These VR systems detected the position of the user and projected immersive, interactive scenes on to the corresponding field of view in stereo. Commercialisation began with the launch of devices, including the Sega VR headset (Horowitz 2004), the VFX1 headset (Chirieleison and Chirieleison, 2004), the Oculus Rift (Luckey 2013), the Google Cardboard (Lyons, 2016), and the HTC Vive (Hilfert and König 2016). Advanced features and functions were developed, including higher resolution wireless headsets, better sensor tracking and an improved user interface (Trentsios et al. 2020).

**Fig. 1:**
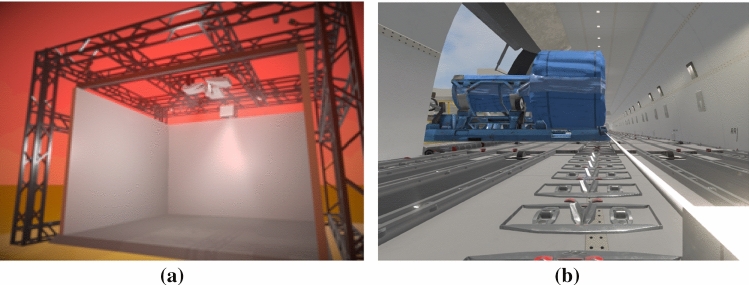
CAVE VR system: **a** system and equipment setup and **b** immersive VR scenes of oversized cargo loading in a 747-8F aircraft

The invention of the CAVE in the early 1990s triggered extensive research to develop user-friendly and low-cost CAVE-like products for teaching, research and industrial applications. The CAVE consists of a cube integrated with screens, 3D projectors, a motion capture system, stereoscopic liquid crystal display shutter-glasses, and computer hardware and software for the VR system. Depending on the field of view, user experience, and system specifications, CAVEs can be built in various forms, such as a cube (North and North 2016; Lau et al. 2009; Czernuszenko et al. 1997), ‘Fishtank’ VR systems (Demiralp et al. 2006), the Star-CAVE (Sterling 2008), the L-shaped CAVE (Zimmermann 2008) and the dome-shaped CAVE (Li et al. 2014). For this study, a CAVE-based system was developed with a cube-like structure, four 3D projectors and screens, audio and acoustic systems, a server, a display system, two high-performance workstations, frame and tracking systems, 3D modelling and VR software and 12 sensors. Our CAVE setup is shown in Fig. 1.

VR has featured in many applications in recent years, including logistics and transport (Wong et al. 2020; Massei et al. 2013), manufacturing (Matsas et al. 2018), product design (Guo et al. 2018), construction (Sampaio and Martins 2014), healthcare (Aiken and Berry 2015), art appreciation (Huang and Han 2014), and education (Müller et al. 2007; Ott and Freina 2015). VR CAVE technology has been applied to teaching and learning in various disciplines, including science (Tarr and Warren 2002; Limniou et al. 2008), engineering (Wang et al. 2018), health care (Bracq et al. 2019), cultural heritage (Vasileva and Petrova 2019), and logistics (Lau et al. 2007). VR facilitates users to understand complex content in an interactive, immersive and multi-dimensional way. For example, through immersing into the designed VR scenes, users can understand material-handling systems in an air cargo terminal, practice crane operations in a container terminal or appreciate ancient art and culture.

The VR CAVE has been applied for years in various disciplines, including education. With the rapid advances in VR technology, will the public accept such new, advanced ways of learning? What are people’s attitudes towards these innovative learning platforms? How effective is learning via VR compared with traditional classroom learning? Most VR research has focused on technological know-how and industrial applications. Few studies have investigated the attitudes, perceptions, and effectiveness of the VR CAVE in an educational context. Increased investigations have been carried out to examine the effectiveness of VR in education until recently, because of the outbreak of the COVID-19 pandemic (Gao et al. 2021; Birrenbach et al. 2021; Kapoor and Singh 2022). The results generally indicated that immersion made a greater contribution than interaction and imagination, whilst the capability of VR interaction aided the development of users’ skills.

Acceptance of VR technology and the effects of personality variables on learning performance are of growing importance as adoption of VR in education and training increases, especially amidst the COVID-19 pandemic. Researchers have used models such as the TAM and the Big Five personality traits to investigate these human interactions and behaviours. Bertrand and Bouchard (2008) investigated the application of the TAM to the use of VR in clinical settings by sampling 141 adults with an interest in VR technology. Kober and Neuper (2013) studied 30 female participants to examine the relationship between personality variables and presence in VR. Their results indicated that absorption, mental imagination, perspective taking, and immersive tendencies showed significant correlations with presence. In contrast to the above research, this study focused on the attitudes and perceived learning behaviour of VR users and how does the users’ openness to IT experience, an internal factor affect these attitudes and perceptions. Responses from over 300 participants were analysed to show the mechanism of how interactive and immersive VR simulation technologies assist learning.

## Hypothesis development

### Enhancing teaching quality and learning experience with VR

VR experience is described as any in which the user is effectively immersed in a responsive virtual world (Brooks 1999). An interactive and immersive environment facilitates teaching and learning, especially in gaining knowledge and practising the skills that are needed in complex operations and are not easily available to learners. Supply chain, transport, and logistics cover sophisticated container operations in aircraft, vessels, cargo terminals, warehouses, distribution centres and retail facilities and involve vast numbers of systems and large amounts of equipment (Burmester et al. 2008). Increasing security often limits the opportunities for onsite learning, meaning VR could be used to provide a virtual environment for learners to understand the end-to-end processes, practise technical skills, and solve simulated problems in sites such as cargo terminals (Lau et al. 2007). Thus, our proposed VR CAVE system would facilitate a deeper understanding of the terminology (e.g., unit load device [ULD], container dolly, and quay crane) by immersing and illustrating trainees operations in a 3D setting. The system would also enable people to gain professional knowledge and skills by experiencing the various functions and flows, such as cargo load planning, container stowage, material handling system operations, and automation of conveyor belts (Martin and Bohuslava 2018). It would also provide a platform for learners to practise technical skills, such as warehouse operations, aircraft cargo loading and container stowage.

The learning effectiveness determines the degree to which learners benefit from VR-assisted teaching and training. These outcomes include knowledge acquisition and skill enhancement. High learning effectiveness can be achieved when learners perceive the technology as useful and easy to use so that they are willing to use it for learning. Therefore, many studies evaluated these users’ perceptions on VR (Akbulut et al. 2018; Chang et al. 2018a, b; Huang and Liaw 2018), which provide directions to further improve the VR scenes and content and enhance teaching and learning experience.

### TAM of VR and engagement in VR-based learning

The TAM (Davis 1989) is a widely accepted theoretical model that is used to predict IT adoption in various contexts. It builds on a wide range of theoretical perspectives and studies, such as the theory of reasoned action (Ajzen and Fishbein 1980), self-efficacy theory (Bandura, 1983), and behavioural decision theory (Beach and Mitchell 1978). The TAM posits that two beliefs—perceived usefulness and perceived ease of use—are the fundamental determinants of use behaviours (Davis 1989; Davis et al. 1989). Based on Davis’ (1989) study, the perceived usefulness of VR and its ease of use are defined as the extent to which a person believes that VR training will enhance his or her learning effectiveness and the extent to which that person believes that VR training will be free of effort.

In addition, the TAM and the TRA both postulate that the two beliefs influence actual behaviours via users’ affective state and/or behavioural intention, and their attitudes towards the use of technology. According to these theoretical models, a person’s affective state, or attitude towards a behaviour, is determined by his or her beliefs about the consequences of exhibiting the behaviour. Davis et al. (1986, p. 987) noted that ‘positively valued outcomes often increase one’s affect towards the means to achieving those outcomes’. Therefore, when a learner perceives that VR training can enhance learning effectiveness, the learner’s affect towards VR training increases. The perceived ease of use also has a positive influence on a learner’s attitudes because of the enhanced self-efficacy and instrumentality. It is to be expected that when VR training is easier to interact with, learners’ efficacy in adopting and operating the system will be greater.

From the perspective of the learning process, encouraging the use of technology is a means of motivating learners’ commitment, involvement, and interaction with learning materials and hence enhancing the effectiveness of their learning. Learning engagement, specifically, the learners’ cognitive and affective engagement (PytlikZillig et al. 2011), is important in the learning process (Fredricks et al. 2004; PytlikZillig et al. 2011), and it serves as the intermediate mechanism between the various ways of training and learning effectiveness. It represents a set of affective and cognitive states that encompass both positive and negative attitudes towards doing the work and the willingness to make the effort to comprehend complex ideas and master difficult skills (Fredricks et al. 2004). Learning engagement depicts affective and cognitive states, but is more relevant in the context of applying technology in learning. For example, Zhang et al. (2017) considered the distinct features of VR on visualisation, interaction, representational fidelity, and immediacy of control that could improve the effectiveness of VR-based learning. Similar studies were carried out by Jou and Wang (2013), Fussell and Truong (2021), and Song et al. (2021). Given the theoretical rationale of the TAM, learning engagement should be influenced by people’s beliefs (i.e. perceived usefulness and perceived ease of use) about VR training.

When learners perceive that VR training helps them to acquire particular knowledge or skills (i.e. perceived VR usefulness) and that the learning process is effortless (i.e. perceived ease of use), they may be more likely to view the potential learning outcomes from VR training positively, and vice versa. According to the TRA (Ajzen and Fishbein 1980), the strength of behavioural belief and the evaluation of the potential outcomes determine the attitude towards the planned behaviour. Therefore, learners with high perceived VR usefulness or high perceived ease of use tend to have a positive attitude towards their learning efforts in VR training. This positive attitude will then motivate learners’ intention to engage themselves in VR-based learning. Therefore, we can expect that the more learners perceive VR training as being useful to their learning and free of effort, the greater their engagement in learning will be. Accordingly, we propose the following hypotheses:

#### H1a

The usefulness of VR that learners perceive has a positive relationship with engagement in VR-based learning.

#### H1b

The ease of use that learners perceive has a positive relationship with engagement in VR-based learning.

Given the relationship between the perception of VR and learning engagement, in the next section, we will move on to discuss how does students’ engagement in VR-based learning affect their learning effectiveness.

### Learning engagement and effectiveness

Many studies have examined learning effectiveness in technology-mediated learning by analysing the influence of engagement on the underlying learning process (e.g. Chen et al. 2010; Russell et al. 2016; Soffer and Nachmias 2018; Guan et al. 2021; Huang et al. 2021). It has been concluded that when learners engage with learning, they spend more time thinking critically and reflectively about the knowledge they acquired and use more higher-order skills, such as problem-solving, collaboration, synthesis, and stimulation, when applying the knowledge (Duderstadt et al. 2002), resulting in greater learning effectiveness (Guan et al. 2021; Huang et al. 2021). In this study, to understand the important role of engagement in VR-based learning, we examined its effect on learning effectiveness, defined as the extent of the knowledge attained in VR training and measured by test results. According to the TRA (Ajzen and Fishbein 1980), stronger behavioural intentions lead to increased effort and a greater likelihood of performing the behaviour. Therefore, if learners are engaged in the VR training experience, the effectiveness of their learning can be expected to be better than that of those who are not engaged because they are more likely to perform the learned activities in the learning process. Accordingly, we propose the following hypothesis:

#### H2

Learners’ engagement in VR-based learning has a positive relationship with learning effectiveness.

In considering the possibility of a joint relationship, H1 and H2 together set the stage for testing the indirect effects of engagement in VR-based learning on the relationship between the TAM of VR and learning effectiveness. Studies have examined the mediating role of learning engagement in the learning process (e.g. Blasco-Arcas et al. 2013; Hu and Hui 2012). Hu and Hui (2012) found that learning engagement mediates the effect of technology-mediated learning on perceived learning effectiveness. In examining the influence on learning performance of active collaborative learning with hyper-interactive teaching technology, Blasco-Arcas et al. (2013) noted the indirect effects of learning engagement. Similarly, the structural equation modelling (SEM) of Huang et al. (2021) also provided empirical support for the mediating role of cognitive engagement in the relationship between users’ VR experience and their learning achievement. This indicates that engagement in learning is an important mediator in explaining the learning process of VR training. Therefore, we propose the following hypotheses:

#### H3a

Learners’ engagement in VR-based learning mediates the positive relationships between the perceived usefulness of VR and learning effectiveness.

#### H3b

Learners’ engagement in VR-based learning mediates the positive relationships between perceived ease of use of VR and learning effectiveness.

As mentioned in the introduction section, learners’ personality affects their learning experience in VR (e.g. Kober and Neuper 2013; Widyanti and Hafizhah 2021). In this study, we further suggest that learners’ openness to experience, a widely studied, learning-related personality in Big Five Inventory, may interact with learners’ perception of VR and affect their learning engagement and effectiveness.

### Moderation of openness to experience on the VR training process

Extending the TRA (Ajzen and Fishbein 1980; Fishbein, and Ajzen. 1975) and the TAM (Davis 1989), many studies have suggested that personality is an important attribute that affects people’s acceptance and use of technology as well as their actual behaviour and performance (Bawack et al. 2021; Devaraj et al. 2008; Wedlock and Trahan 2019). A meta-analytical study also found that attitude and personality tend to moderate behavioural intention (Armitage and Conner 2001; Devaraj et al. 2008). Building on these studies, the moderating roles of personality traits were tested by examining the extent to which the TAM predictors resulted in learners’ engagement and learning effectiveness in VR training.

Among the personality traits in the Big Five Inventory (Costa and McCrae 1992), people with greater openness to experience tend to adopt an open attitude and are more willing to try new and different things (Madrid and Patterson 2016). These people are more imaginative, broad-minded, curious, and unconventional, all of which are attributes associated with positive attitudes and motivation for learning (Devaraj et al. 2008; Madrid et al. 2014). Openness to experience has also been conceptualised as the tendency of an individual to favour innovation, exploration, and diversity over convention (Matz 2021; McCrae and Sutin 2009). A meta-analysis conducted by Barrick and Mount (1991) supported this view by identifying the relationship between openness to experience and training proficiency (i.e. learning effectiveness in training).

In this study, we propose that openness to experience plays an important role in reinforcing the effect of the TAM on learning engagement and thus learning effectiveness in VR training. In particular, the connection between a high level of acceptance of VR training and engagement, as well as learning effectiveness, is strengthened when individuals are highly open to new experiences. Individuals with a high level of openness to experience are more willing to try new and different things, tending to seek out new and varied experiences and embracing change (Matz 2021; Puente-Díaz et al. 2022). Therefore, they are more self-motivated to engage in a new VR training experience based on their positive perception of its usefulness and ease of use, thus facilitating their learning effectiveness. In contrast, although those with a low level of openness to experience perceive VR training as useful and easy, they still have doubts and resist experiencing VR-based learning, leading to reduced engagement and thus weaker learning effectiveness. Accordingly, openness to experience serves as a critical reinforcer that strengthens individuals’ learning engagement when they receive VR training. To test the conditional effect of openness to experience on the VR-based learning process, we specifically examined the moderating effect of openness to experience on the relationship between the TAM of VR and learning effectiveness as mediated by learning engagement:

#### H4a

Learners’ openness to experience moderates the mediating effect of engagement in VR-based learning on the relationship between learners’ perceived usefulness of VR and learning effectiveness.

#### H4b

Learners’ openness to experience moderates the mediating effect of engagement in VR-based learning on the relationship between learners’ perceived ease of use of VR and learning effectiveness.

**Fig. 2:**
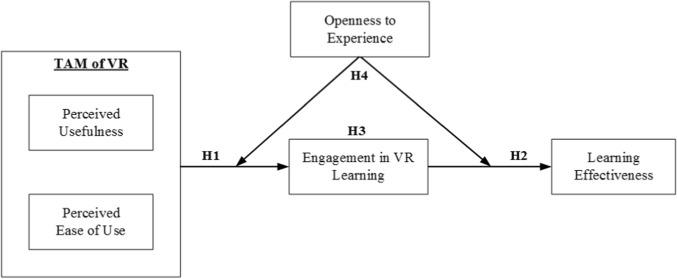
Summary model of hypothesised relationships. (H = Hypothesis)

The hypothesised model is summarised in Fig. 2.

## Methodology

### Research design

**Fig. 3:**
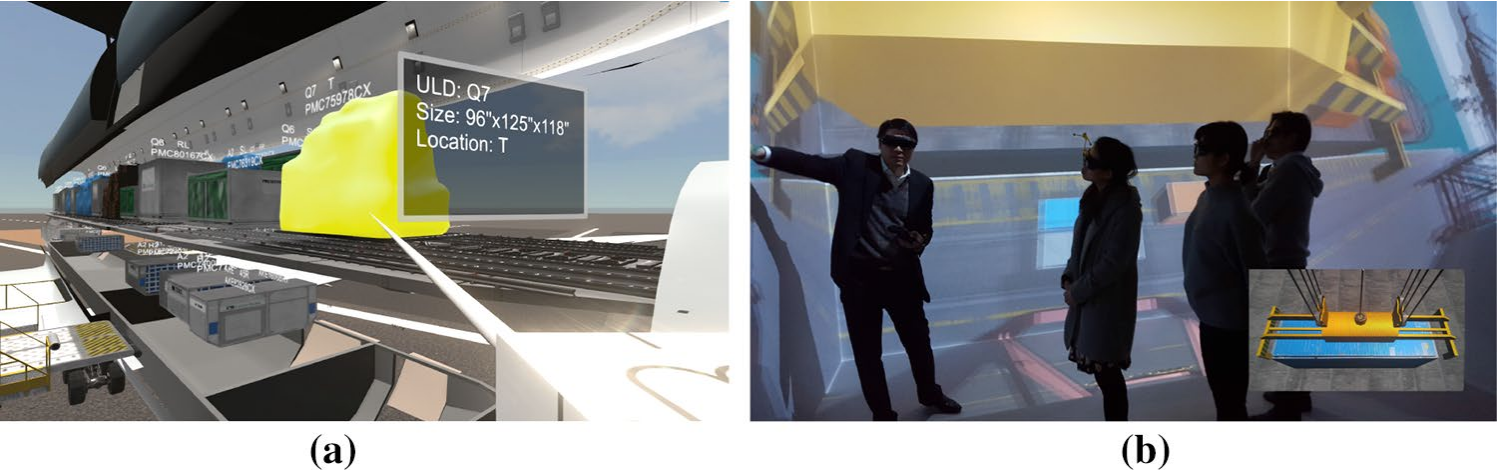
Use of VR to explain and illustrate the functions of facilities in **a** air cargo terminal and **b** quay crane operations in a container terminal

This study evaluated learners’ openness to experience, as well as their perceived usefulness of, perceived ease of use of, and engagement in VR-based learning and the learning effectiveness of adopting VR for training. The study first developed and established a VR CAVE system with three interactive and immersive logistics and transport scenes for teaching and learning. The VR platform with simulation scenes included training on air cargo terminal operations, aircraft cargo loading and maritime port operations. The learning content of the three VR scenes in the training covered an air cargo container that is a ULD, passing through an air cargo terminal, moving from inbound to the terminal, through weighing and customs clearance, storage in material handling systems, transport in a dolly, to loading into an aircraft. Figure 3a shows how VR was used to illustrate the cargo load planning of ULD on the main deck of a 747-8F airfreighter. The ULD and oversize cargo loading operations in an aircraft were then illustrated and explained. The training was then extended to port terminals, with users operating the container loading and discharge operations of a terminal quay crane in a simulated immersive environment (Fig. 3b). The principles of container stowage planning on the vessels were explained with the use of interactive VR scenes.

A pilot study was conducted to examine the model on the interaction of openness to IT experience and the perception of VR-based learning before a comprehensive survey was undertaken. To test the model, a survey was conducted. The sample of 175 respondents who participated in the VR training was divided between students (69%) and full-time practitioners (31%). 2% were younger than 18 years, 69% were between 18 and 25 years, 11% were between 26 and 35 years, 6% were between 36 and 45 years, 10% were between 46 and 60 years, and 2% were older than 60 years. The participants were asked to complete the survey after completing the learning experience on cargo terminals and aircraft operations using the VR platform.

The survey examined three key constructs—openness to IT experience (three items; *α* = 0.88), perception of VR training (five items; *α* = 0.90) and perceived learning effectiveness (three items; *α* = 0.94)—and two demographic variables (i.e. age and occupation). Simple regression showed that both openness to IT experience (*β* = 0.45, *p* < 0.01) and perception of VR training (*β* = 0.47, *p* < 0.01) were positively associated with the perceived effectiveness of learning. However, their interactive effect on the perceived effectiveness of learning was not significant (*β* = − 0.02, *ns*). It was concluded from the pilot study that the design of VR training was appropriate, but the measure of learning effectiveness and the perception of VR training needed improvement. For example, pre-training and post-training performance tests should be incorporated, including multiple-choice questions about the training content, to measure the participants’ knowledge before VR training and the actual learning effectiveness after it. Furthermore, the general perception of VR training should be divided into two specific dimensions, namely the perceived usefulness of VR training and perceived ease of use, based upon the TAM (Davis 1989).

After reviewing the results of the pilot study, a comprehensive cross-sectional survey with 92 questions across six sections was compiled, with a measure of five constructs of psychometric properties, focusing on undergraduate business students. The final sample in this study consisted of 321 undergraduate students at a self-financed university in Hong Kong. The mean age was 20.51 years (SD = 1.00). Most of the students were studying management, supply chain management, and business administration. Forty-nine per cent of the students were majoring in Management, 40.50% of them are studying Supply Chain Management, 6.85% of the students are studying business administration, including Marketing, Accounting, and Finance. The remaining students fall into other disciplines, such as Translation and Journalism. The sample was analysed after the participants completed a questionnaire about their demographic characteristics. The results are shown in Table 1.

**Table 1:** Demographic characteristics of the sample

Characteristics variables	Features	Sample distribution (%)	Number of respondents (*n*)
Gender	Male	34.00	109
	Female	66.00	212
Age	≤ 19	6.85	22
	20	55.76	179
	21	25.54	82
	≥ 22	11.83	38
Major studies	Supply chain management	40.50	130
	Management	49.22	158
	Business administration	6.85	22
	Others	3.43	11

The training with the VR platform was part of the curriculum in the students’ modules. To assess their knowledge of the subject matter (i.e. supply chain and logistics), the participants were invited to complete a survey measuring their openness to experience (10 items) and a pre-training performance test (five questions) before taking the training with the VR platform. After the VR training, the participants were asked to complete the second part of the survey, which was designed to measure the TAM of VR (i.e. perceived usefulness of VR [four items], perceived ease of use [four items], engagement in VR-based learning [seven items], learning effectiveness [eight questions], and respondents’ demographics, including gender, age, and major of study). The survey was conducted in English.

### Research measures

Unless otherwise noted, a 7-point scale (1 = not at all, 7 = to a very great extent) was used in each of the questionnaires described below.

*Openness to experience*. Before the VR training, the participants were asked to assess their openness to experience with items adopted from John and Srivastava (1999). The measure consisted of 10 items, such as ‘I see myself as someone who is original and comes up with new ideas’. Cronbach’s alpha for the measure was 0.81.

*Perceived VR usefulness (PU) and perceived ease of VR use (PEU).* Both PU and PEU consisted of four items, which were adapted from Davis (1989) and Davis et al. (1989) to reflect the VR training context. An example of a PU item was ‘I find VR is useful in my study’ (alpha = 0.93), and a PEU example was ‘Interacting with VR does not require a lot of mental effort’ (alpha = 0.88).

*Engagement in VR-based learning.* The participants were asked to report their commitment to, involvement with, participation in, and interaction with VR technology using a seven-item measure adapted from PytlikZillig et al. (2011) (e.g. ‘VR technology helps me gain a deeper understanding of the concepts presented in class’; alpha = 0.85).

*Learning effectiveness.* The learning effectiveness of VR training was measured by eight multiple-choice questions about the content after the VR training. A sample question was ‘What is the function of dollies in the air cargo terminal? A. To transport passengers to the rampside of the airport; B. To load, transport and unload ULD and cargo pallets; C. To temporarily store unit-load devices and cargo pallets; D. To supply electricity to the movable platform at the rampside of the airport; E. To transfer livestock to the aircraft’. The questions were all designed to assess the knowledge of supply chains, operations management, and logistics that the students had gained via immersion and interaction with the VR scenes. We marked the scores based on the number of correct answers, and the scores ranged from 0 to 8.

*Control variables*. All of the participants were invited to test their basic knowledge of the training content before starting the VR training. The participants’ pre-training test performance, like the evaluation of the learning effectiveness of VR training, was assessed using five multiple-choice questions about the content. The score reflected the number of correct answers to the five questions (from 0 to 5). Other control variables were age and gender. Age was a continuous variable, whereas gender was dummy-coded (female = 0; male = 1).

## Results

### Descriptive statistics of study variables

Table 2 displays the means, standard deviations, internal consistency reliabilities, and bivariate correlations for the measures used in this study.

**Table 2:** Descriptive statistics and correlations

Variables	M*	SD*	2	3	4	5	6	7	8	9
Perceived usefulness	5.03	1.00	(.93)							
Perceived ease of use	4.77	.97	.64**	(.88)						
Age	20.51	1.00	.05**	.04**	–					
Gender^a^	.70	.46	−.14**	−.17**	−.19**	–				
Pre-training test performance	3.45	1.21	.03**	.04**	.01**	−.03**	–			
Openness to experience	4.45	.79	.30**	.22**	.06**	−.19**	−.05*	(.81)		
Engagement in VR-based learning	5.06	.97	.65**	.48**	−.02**	−.08**	.05**	.18**	(.85)	
Learning effectiveness	5.43	2.00	.21**	.11**	−.14**	.05**	.21**	−.02**	.27**	–

*n* = 321 (listwise)

^a^Dichotomous variable (0 = Male, 1 = Female)

*p < .05, ** *p* < .01, two-tailed

### Hypotheses testing

**Fig. 4:**
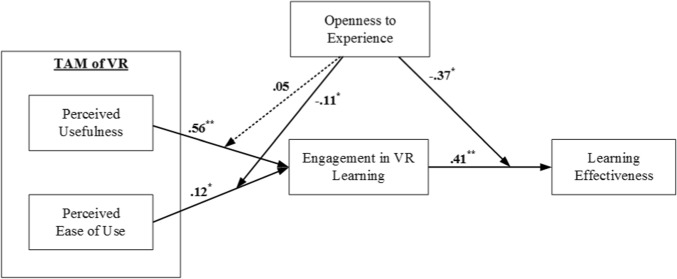
Standardised parameter estimates for the model

Overall, the hypothesised model was a good fit for the data (*χ*^2^(18) = 38.15, *p* < 0.01, CFI = 0.98, NFI = 0.96, IFI = 0.98, RMSEA = 0.05). Moreover, when the proposed moderation effects were removed, the overall fit deteriorated significantly (*χ*^2^_diff_(11) = 20.01, *p* < 0.05, CFI = 0.95, NFI = 0.94, IFI = 0.95, RMSEA = 0.10). The individual hypotheses are now addressed (see Table 3 and Fig. 4).

**Table 3:** Structural equation findings for the hypothesised model (H1–H4)

Variables	Engagement in VR-based learning	Learning effectiveness
Control variables		
Age	−.05**(.04)	−.22**(.11)
Gender	.02**(.09)	.15**(.23)
Pre-training test performance	.02**(.03)	.33**(.08)
Key variables		
Perceived usefulness of VR (PU)	.56**(.06)	.28**(.16)
Perceived ease of use of VR (PEU)	.12**(.06)	−.10**(.14)
Openness to experience (OE)	−.02**(.05)	−.17**(.13)
Engagement (EN)		.41**(.14)
OE * PU	.05**(.06)	.36* (.18)
OE * PEU	−.11**(.07)	−.38* (.16)
OE * EN		−.38* (.17)

*n* = 321. Standardised estimates (based on grand-mean centring) are reported, with standard errors in parentheses

*p < .05, two-tailed. ** *p* < .01, two-tailed

H1, covering the direct effect of the TAM of VR on engagement in VR-based learning, was tested with SEM, with age and gender as covariates. The result of SEM showed that perceived usefulness of VR (*β* = 0.56, *p* < 0.01) and perceived ease of use (*β* = 0.12, *p* < 0.05) had a positive relationship with engagement in VR-based learning. Furthermore, in line with H2, engagement in VR-based learning showed a positive relationship with learning effectiveness (*β* = 0.41, *p* < 0.01); therefore, H1 and H2 were both supported.

Consistent with the criteria of Shrout and Bolger (2002), support for H1a, H1b and H2 leaves open the possibility that engagement in learning mediates the relationship between the TAM of VR (i.e. perceived usefulness of VR and attitude towards VR) and the learning effectiveness of VR training. Table 4 shows the direct and indirect effects (Preacher and Hayes 2004, 2008). In line with H3a, engagement in VR-based learning mediated the relationship between the perceived usefulness of VR and learning effectiveness; the 95% confidence interval (CI) (0.10 to 0.51) associated with the indirect effect (*β* = 0.28, *p* < 0.01) excluded zero. Furthermore, in support of H3b, engagement in VR-based learning mediated the relationship between the perceived ease of use and learning effectiveness; the 95% CI (0.13 to 0.43) associated with the indirect effect (*β* = 0.24, *p* < 0.01) excluded zero. Finally, engagement in VR-based learning accounted for 12.25% of the variance in the perceived usefulness of the VR-learning effectiveness relationship and 12.02% of the variance involving the perceived ease of use and learning effectiveness.

**Table 4:** Engagement in VR-based learning as a mediator of the TAM of VR-learning effectiveness relationships

Bootstrap estimate	TAM of VR^#^			
	Perceived usefulness		Perceived ease of use	
	*β*	SE	*β*	SE
Path analysis				
TAM of VR^#^ –EN (a path)	.63**	.05	.47**	.05
EN–LE (b path)	.45**	.15	.52**	.13
Total effect (c path)	.41**	.11	.25**	.12
TAM of VR^#^ –LE (c’ path)	.13**	.15	.01**	.13
Bootstrapping				
Indirect effect	.28**	.10	.24**	.07
Bias Corrected 95% CI (Lower–Upper)	.10**	.51	.13**	.43
*F*	7.90**		7.73**	
*R*^*2*^	.12**		.12**	
*ΔR*^*2*^	.11**		.10**	

TAM of VR = technology acceptance model of virtual reality; EN = engagement in VR-based learning; LE: learning effectiveness; SE = standard error; CI = confidence interval based on 1000 bootstrap samples

# Results for perceived usefulness of VR are in the left column, and in the right column for attitude to VR

*p < .05, two-tailed. **p < .01, two-tailed

Finally, the moderated mediation models predicted in H4 were tested with the approach used by Preacher et al. (2007) (cf. Ng et al. 2008). We used PROCESS model 58, a macro for SPSS that conducts observed-variable moderated mediation analysis (Hayes 2018) to examine the model. This requires the magnitude of the moderated conditional indirect effect of the TAM of VR on learning effectiveness via engagement in VR-based learning to differ across high and low levels of the moderator (i.e. openness to experience). The SEM result showed that openness to experience moderated the positive relationship between the perceived ease of use and engagement in VR-based learning (*β* = −0.11, *p* < 0.05), but not the relationship between the perceived usefulness and engagement in VR-based learning (*β* = 0.04, *ns*). Therefore, H4b was examined, but not H4a.

**Fig. 5:**
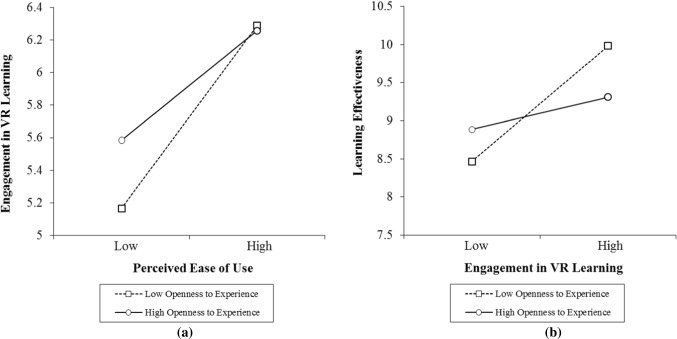
Moderation effects of openness to experience on **a** perceived ease of use-engagement in VR-based learning and **b** engagement in VR-based learning–learning effectiveness relationships

For H4b, a moderated mediation effect was found when the interaction between openness to experience and perceived ease of use (*β* = −0.15, *p* < 0.05) and the interaction between openness to experience and engagement in VR-based learning (*β* = −0.37, *p* < 0.01) moderated the indirect effects of the perceived ease of use on learning effectiveness via engagement in VR-based learning (*β* = 0.50, *p* < 0.01) (see the left-hand side of Table 5). The bottom half of Table 5 (left-hand side) shows that the indirect effect was statistically significant at the mean level (i.e. average openness to experience) and at one standard deviation above the mean (i.e. high openness to experience). This means that engagement in VR-based learning mediated the effects of the perceived ease of use on learning effectiveness when the participants’ openness to experience was average to high. At one standard deviation below the mean level (i.e. low openness to experience), the indirect effect was not statistically significant, which means that engagement in VR-based learning did not mediate the effects of the perceived ease of use on learning effectiveness when the participants’ openness to experience was low. It was concluded that learners with a moderate-to-high level of openness to experience who perceived VR as easy to use therefore showed greater engagement in VR-based learning than those with average to high openness to experience who perceived VR as being difficult to use. However, for learners with low openness to experience, engagement in VR-based learning did not mediate the effects of the perceived ease of use on learning effectiveness (see Fig. 5).

**Table 5:** Moderated mediated results for learning effectiveness across levels of openness to experience (for PEU of VR)

	Engagement in VR-based learning			Learning effectiveness		
	*β*	*SE*	*t*	*β*	*SE*	*t*
*Control variables*						
Age	−.03**	.05**	−.69**	−.23**	.11**	−2.13**
Gender	−.00**	.11**	−.06**	.05**	.25**	.22**
Pre-training Test Performance	.00**	.04**	.06**	.31**	.09**	3.32**
*Key variables*						
Perceived Ease of Use of VR (PEU)	.46**	.05**	8.74**	.00**	.13**	.04**
Openness to Experience (OE)	.12**	.07**	1.85**	.77**	.72**	2.45**
*Moderation effects*						
OE * PEU	−.15**	.06**	−2.50**			
OE * EN				−.37**	.14**	−2.63**
Mediation effects						
Engagement in VR-based learning (EN)				.50**	.13**	3.92**
*ΔR*^2^	.02**			.02**		
*R*^2^	.25**			.14**		

*Moderator*	Conditional Indirect effect	*SE*	*Z*	Conditional Indirect effect	*SE*	*Z*
− 1 *SD* OE (−.75)	.57**	.07**	7.87**	.78**	.16**	4.92**
Mean OE (0)	.47**	.05**	8.81**	.52**	.13**	4.08**
+ 1 *SD* OE (.75)	.35**	.06**	5.46**	.23**	.17**	1.33**

*p < .05. **p < .01

Furthermore, a moderated mediation effect was also found when the interaction of openness to experience with engagement in VR-based learning (*β* = −0.37, *p* < 0.01) moderated the indirect effect of the perceived usefulness of VR on learning effectiveness via engagement in VR-based learning (*β* = 0.40, *p* < 0.01) (see the right-hand side of Table 5). As shown in the bottom half of Table 5 (right-hand side), the indirect effect is statistically significant at the mean level (i.e. average openness to experience) and at one standard deviation above the mean (i.e. high openness to experience). This indicates that engagement in learning mediated the effects of the perceived usefulness of VR on learning effectiveness when the participants’ openness to experience was average to high. At one standard deviation below the mean level (i.e. low openness to experience), the indirect effect was not statistically significant, which means that engagement in VR-based learning did not mediate the effects of the perceived usefulness of VR on learning effectiveness when the participants’ openness to experience was low. It was concluded that learners with a moderate-to-high level of openness to experience who perceived VR as useful show greater engagement in VR-based learning than those with an average-to-high level of openness to experience who perceive VR as not useful. However, for learners with a low level of openness to experience, engagement in VR-based learning did not mediate the effects of the perceived usefulness of VR on learning effectiveness (see Fig. 5). Overall, H4b was supported, but H4a was not.

## Discussion and implications

This study enriches the knowledge of what determines the learning effectiveness of VR training, especially in an educational setting. Our study presents a novel pedagogical conceptualisation of learning the management of complex operations, supply chains, and logistics using an immersive and interactive VR platform. Based on the TAM3 (Davis 1989; Venkatesh and Bala 2008), the findings advance our understanding of the adaptation process of VR training by first exploring the effects of an individual intrinsic factor.

(i.e. openness to IT experience) on the perceived usefulness and ease of use of VR training and thus the individual’s attitude towards learning. Exploring these relationships also helps bridge the gap inherent in using theories of management psychology to explain the effectiveness of technology-based learning. As scholars have noted, theories about learning and psychology have rarely been considered in developing VR applications to enhance learning outcomes (Radianti et al. 2020). The literature on the Big Five personality traits has been incorporated into the TAM3 to regulate the level of engagement in learning and its consequent learning outcomes. Building on the TAM3, we specifically examined the moderating role played by a major individual trait in the mediation relationship and revealed that individuals with a greater willingness to accept new experiences are better able to adapt to new forms of learning, such as VR technology-mediated training. This finding is consistent with previous studies of the Big Five personality traits in the management literature, in which openness to experience is crucial in determining an individual’s learning effectiveness (Chow 2018; DeYoung et al. 2014; Kaufman et al. 2010).

The present study also examined the important role of the perceived usefulness of VR training in the learning process. According to the TAM3, the perception of the usefulness of technology mediates the relationship between individual differences and behavioural intention in learning. Furthermore, Calisir et al. (2014) and Venkatesh and Bala (2008) indicated that perceived usefulness is the strongest predictor of the behavioural intention to adopt a new learning practice. In this study, the perceived usefulness of VR training helped explain why people with a high level of openness to IT experience learned better than those with a low level of openness to IT experience. Thus, it was found that because people with a high level of openness to IT experience held a more positive view of VR training, they engaged more in the learning process of VR training, which in turn led to a more positive perception of learning, particularly in a specific subject matter.

The findings of this study have valuable implications for educators, corporate trainers, and managers about using VR training to develop human resources. First, to maximise the learning effectiveness of VR training, it is recommended that teachers and trainers prepare learners by ensuring they have an open mindset before the training event. Jackson et al. (2012a, b) demonstrated that openness to experience can be enhanced through cognitive training, such as instruction in inductive reasoning, crossword and Sudoku puzzles, and coaching (Hui et al. 2013, 2019). Enhancing learners’ openness to experience through these cognitive training would strengthen learners’ engagement in learning which further leads to better knowledge acquisition and skills development in VR training. Second, educators, corporate trainers, and managers should brief trainees on the value and usefulness of VR training. They should explain how the content relates to their studies, to their work, and to attaining their goals, and how VR training can benefit their learning effectiveness when compared with traditional methods, especially in working environments that involve tough safety and security requirements or sophisticated operations procedures. A briefing can enhance the usefulness learners perceive in the VR training, thus heightening their engagement and the effectiveness of their learning.

## Conclusion

The increasing use of VR technology and its applications in education obliges educators to understand the effectiveness of training using VR and the challenges to adopting VR systems, as well as the importance of user acceptance and attitudes towards new pedagogical teaching methods. In this study, we used the novel pedagogical development of VR immersive and interactive scenes to illustrate the cargo loading operations of aircraft, an air cargo terminal, and a port terminal. The results showed that individuals’ intrinsic factors, including openness to IT experience, influenced their perceived usefulness of VR training and attitudes towards learning. With reference to the TAM3, by measuring learners’ attitudes towards the use of VR in learning, we revealed that the participants who were open to new experiences in the use of IT scored higher in the usefulness they perceived in VR training and showed a more positive attitude towards learning. The findings were also consistent with the Big Five personality management theories, in which openness to experience is a crucial personal trait that determines an individual’s learning effectiveness.

The findings of this study should be considered in light of several major limitations. Given our limited resources, the training content in the VR platform was developed containing several important scenes of cargo operations but did not reflect everything that can occur in a working environment. More examples of how to handle exceptions could be incorporated into the platform to help users understand and practise the required skills. Another limitation was that most of the respondents were young. Further studies could be carried out with a wider age range. In the survey, most of the data were collected at the same time and the cross-sectional design did not permit the examination of causality among the variables. Accordingly, future research could address the question of causality by examining personality, perceptions, behaviours, and learning effectiveness in a longitudinal setting.

This study focused exclusively on one of the Big Five traits—openness to experience—and examined its effect on the learning effectiveness of VR training. Research has indicated that other Big Five traits are also related to learning (e.g. Katrimpouza et al. 2019; Tabatabaei et al. 2018). Further research could explore whether individual differences, such as other Big Five traits (i.e. conscientiousness, emotional stability, extraversion, and agreeableness) (Busato et al. 1998; Komarraju et al. 2011), moderate the learning effectiveness of VR training (Chang 2005; Haji et al. 2016). Future studies could examine the use of VR in learning how to act in complex problem-solving situations in cargo terminal and aircraft load planning operations. Furthermore, the literature has shown that situational factors, such as the complexity of the learning task and learners’ expertise (Hui et al. 2013, 2019), may moderate learning effectiveness. Therefore, future research could also explore the effects of different situational factors on the learning effectiveness of VR training, as well as of individual differences. The complexity of VR-based learning could also be enhanced with the further grade of VR systems that could support collaborative and team learning.

## Data Availability

The datasets generated during and/or analysed during the current study are available from the corresponding author on reasonable request.
